# Cattle production, water resources, and sustainability: a review of current knowledge and future directions

**DOI:** 10.1093/af/vfag041

**Published:** 2026-10-19

**Authors:** Kevin Wagner, Susan Metzger, Brent Auvermann

**Affiliations:** Oklahoma State University, Stillwater, OK; Kansas State University, Manhattan, KS; Texas A&M University, Amarillo, TX

**Keywords:** beef water footprint, feedyard water use, grazing management, research needs, water quality

Implications

- Water quality degradation, increasing drought severity, and low BMP adoption threaten grazing systems, but riparian protection, adaptive grazing, precision livestock technologies, and coordinated Extension–Natural Resources Conservation Service support can meaningfully improve resilience and water outcomes.
- Feedyards face parallel challenges, particularly nutrient‑laden runoff, high indirect water use for feed, and declining irrigation supplies, yet innovations in manure management, wastewater reuse, and low‑water forage systems offer viable pathways to reduce water demand.
- Significant knowledge gaps remain, including limited watershed‑scale data, insufficient evaluation of technologies, and the need for practical reuse strategies and drought‑tolerant feed crops.
- Long‑term water sustainability requires systems‑level approaches, improved incentives, and policy alignment, as technological efficiency alone cannot overcome economic and operational barriers to widespread adoption.

## Introduction

Cattle production is a cornerstone of U.S. agriculture, but it faces growing scrutiny regarding sustainability. A key driver of industry adoption of more sustainable production systems is consumer demand for environmentally responsible food products, along with evolving opportunities for market differentiation (Ishaq et al., 2024). This review evaluates water-related challenges in grazing and feedlot systems, synthesizes current research on watershed impacts and the beef water footprint, and highlights emerging strategies to support more sustainable animal production. It identifies best management practices (BMPs) for enhancing water resources, collaborative strategies for water stewardship, and critical knowledge gaps. By mapping existing studies and identifying priority areas for further investigation, this article aims to draw a cohesive roadmap for scalable interventions to enhance water sustainability in US beef production.

## Water Resource Challenges and Solutions in Cattle Production Systems

### Grazing systems

The upstream value chain within the domestic beef-production system in the United States features a wide variety of extensive components (Heflin et al., 2019) wherein cattle graze native rangeland, native pastures, and/or modified pasture, thereby taking advantage of the ruminant’s capacity to convert relatively low-digestibility plant materials into animal protein and fat. Those rangelands and pastures may nourish beef cattle at any growth stage, including unweaned and weaned calves, stocker cattle, and even finishing animals, depending on the interaction of market conditions with forage availability and quality. Although well over 90% of the domestic beef slaughter consists of animals that have spent some time in a confinement system (aka “feedyard” or “feedlot”) (ERS, 2025), nearly all such animals have spent a substantial proportion of their lives on some kind of grazing resource. Indeed, beef cattle are grazed throughout the entire United States on resources of highly variable quality and productivity in both space and time, but the contemporary interest in the carbon footprint of domestic beef production has focused attention on the Great Plains ecosystem, where grazing beef cattle have largely displaced native American Bison (Allred et al., 2011; De Bernardi et al., 2025) since the late 1800 s.

Challenge: Direct cattle access to surface waterbodies can lead to water quality issues like nutrient loading, pathogen contamination, bank destabilization, erosion, and degradation of riparian and aquatic habitats (Agouridis et al., 2005, Figure 1). Overgrazing of upland areas further exacerbates nutrient, sediment, and bacterial runoff to nearby waterbodies. Bacterial source tracking studies in Texas indicate that cattle and other domestic animals contribute 14–29% of bacterial loading in impaired waterbodies (Di Giovanni et al., 2013). The Oklahoma DEQ identifies “riparian grazing” and “rangeland grazing” as two of the top five sources of lake and river impairment for the state (Phillippe, 2024). These issues have resulted in Total Maximum Daily Loads (including the high profile Chesapeake Bay TMDL in Maryland, Virginia and surrounding states, and Tomales Bay Pathogen TMDL in California) and watershed based plans (including the Lake Okeechobee Basin Management Action Plan in Florida and Lavaca River Watershed Protection Plan in Texas) frequently identifying cattle as major contributors to pollution in the Midwest, West, Rocky Mountains, Southwest, and South, and recommending implementation of grazing and riparian protection BMPs (Agouridis et al., 2005).

**Figure 1.**
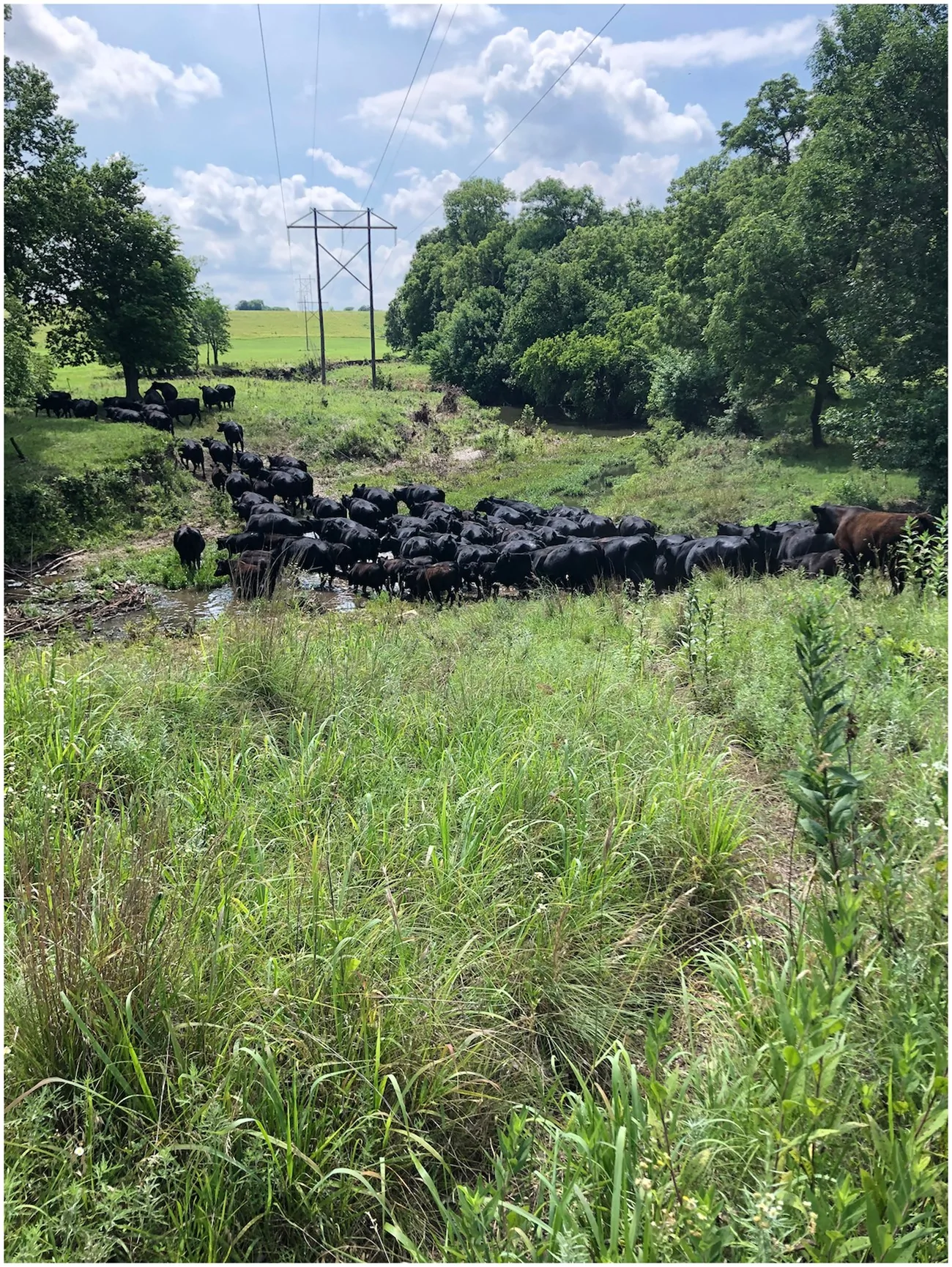
Cattle with access to streams can cause bank erosion and increase nutrient loading in the waterways.

Challenge: Drought has long posed a major challenge to cattle production. Reduced precipitation lowers the quantity and quality of available forage, increases reliance on supplemental feed, diminishes animal condition, and triggers costly destocking–restocking cycles, which elevates operating expenses and reduces overall profitability. Furthermore, soil infiltration and water retention are widely recognized as primary constraints in grazing land ecosystems (Teague et al., 2011). In regions dependent on groundwater, these impacts may be compounded by declining aquifer levels and water scarcity, which can reduce both the availability and quality of water for grazing and feeding operations (Theurer et al., 2026). Historically, U.S. cattle numbers declined during major droughts but recovered by subsequent agricultural censuses. However, the 2010s drought, often viewed as indicative of future extreme drought conditions, resulted in substantial regional cattle inventory declines. Relative to the 2007 census, cattle numbers declined 17.6% in the Southern Plains (Klemm and Briske, 2021). Before cattle numbers rebounded, the 2020 to 2025 drought reduced cattle numbers to their lowest since the 1950s, forcing packing plant shutdowns and reduced shifts. As droughts increase globally, producers are seeking management practices to enhance resilience.

Challenge: Limited adoption of improved grazing systems may stem from knowledge gaps or insufficient economic incentives. Forage responses to improved grazing systems often require several years to reach full production potential which delays returns on producer investment and reduces the practices’ near‑term economic appeal (Dyer et al., 2021). Further, improved grazing practice adoption is often limited by the need for additional fencing, increased labor, and reliance on leased land. High fencing costs, the inflexibility of permanent structures, and the difficulty of installing fences in floodplains discourage landowners from implementing measures like riparian protection and rotational grazing systems. As a result, only 25% of cattle operations in the Southern Plains employ rotational grazing systems (Murray et al., 2025).

Solution: Practices supporting riparian protection (riparian fencing, off-stream water, shade structures, limited riparian grazing, etc.) have been shown to effectively reduce sediment and fecal bacteria by mitigating riparian vegetation trampling, in-stream defecation, and streambed disturbance from cattle (Grudzinski et al., 2020). Relative to direct stream deposition, manure deposited 0.6 m and 2.1 m from a stream reduced bacterial inputs by 83% and 95%, respectively (Larsen et al., 1994). Targeted grazing of riparian pastures has been proposed as a mitigation strategy, as *E. coli* concentrations were 88% lower when runoff occurred more than 2 wk after grazing, compared with runoff during or immediately following grazing (Wagner et al., 2012). This suggests that timing riparian grazing with ­periods of low rainfall risk may reduce contamination.

Solution: Grazing practices that restore soil function by increasing ground cover and soil carbon have been proposed to enhance drought resilience by improving infiltration and soil water‑holding capacity, thereby accelerating recovery following droughts and pluvial disturbances (Teague and Kreuter, 2020; Dyer et al., 2021). Furthermore, grazing systems that improve manure distribution and vegetative cover can also enhance infiltration, runoff filtration, and water quality when used in conjunction with moderate stocking rates (Briske et al., 2011).

Several rotational grazing systems (e.g., adaptive multi‑paddock [AMP], prescriptive, pyric herbivory) have been developed as alternatives to continuous grazing. Empirical and modeling studies indicate reduced runoff and pollutant losses under these systems relative to continuous grazing. Wagner et al. (2023) reported 24% greater edge-of-field runoff and significantly higher nitrogen and sediment losses under continuous grazing compared with prescribed grazing. Ranch‑scale APEX model simulations suggest AMP grazing reduces runoff, sediment, and nutrient losses by 44% to 48% relative to light continuous grazing, and 142% to 158% relative to heavy continuous grazing (Park et al., 2017). Similarly, watershed-scale SWAT modeling showed consistent reductions in hydrologic losses and pollutant export when shifting from heavy continuous to AMP grazing (Park et al., 2017; Kim et al., 2023; Figure 2).

**Figure 2.**
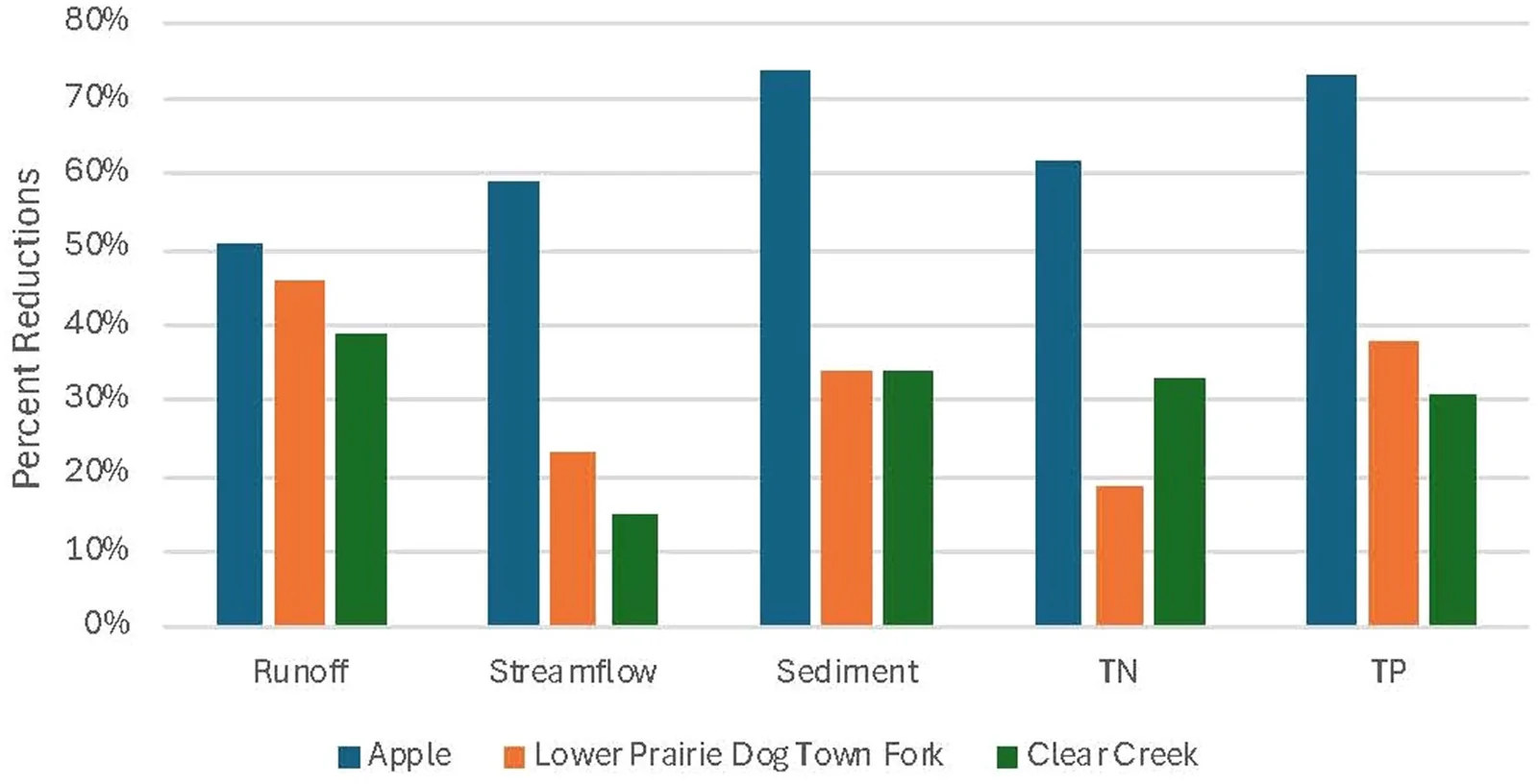
Simulated runoff, streamflow, sediment, total nitrogen (TN) and total phosphorus (TP) reductions from conversion from heavy continuous grazing to AMP in the Apple (North Dakota), Lower Prairie Dog Town Fork (Texas), and Clear Creek (Texas) watersheds.

Despite these trends, ecosystem responses to AMP grazing are not uniform. Whereas Hillenbrand et al. (2019) observed significantly higher infiltration under AMP than heavy continuous grazing in South Dakota, Teague et al. (2011) found no treatment differences in infiltration or hydraulic conductivity across four Texas ranches under heavy continuous, light continuous, and AMP grazing. However, runoff measured via rainfall simulator was significantly lower only under light continuous, whereas sediment loss was significantly higher only under heavy continuous. Soil moisture was also significantly lower under heavy continuous compared with all other management treatments (Teague et al., 2011). Collectively, this suggests that while AMP grazing can enhance hydrologic function and water quality, outcomes depend on stocking rate, climate, and site‑specific soil conditions.

Solution: Precision livestock technologies provide real-time data, enabling ranchers to take timely action. New technologies like virtual fencing (VF) offer a promising alternative to help address adoption barriers and improve the feasibility of implementing adaptive grazing management by reducing the need for physical fences and the labor demands of manual herd movement. Short-term trials in Oklahoma suggest VF is effective for managing cattle, with cattle compliance with exclusion zones exceeding 80% (Murray et al., 2025). In addition to VF, wearable sensors and tags that track cattle grazing and remote sensing using drones and satellite imagery to monitor forage biomass and quality offer new opportunities to enhance grazing management and sustainability (Marchegiani et al., 2025).

Solution: Coordination between Extension educational programming and USDA Natural Resources Conservation Service (NRCS) technical and financial support can strengthen adoption and long‑term effectiveness of BMPs (Peterson et al., 2015). Peterson et al. (2015) examined factors influencing conservation practice adoption among Texas beef cattle producers and identified engagement with Extension services as the strongest predictor of adoption. Similarly, Dewald et al. (2019) found that landowners identified Extension as the most trustworthy source of water related information in Texas.

Financial incentives can influence adoption of prescribed grazing, but their effectiveness depends strongly on ecological and economic context. Dyer et al., (2021) showed that producer returns from soil‑health–oriented grazing practices depend on forage response dynamics, initial rangeland condition, implementation costs, and producer discount rates. When delayed productivity gains and establishment costs are considered, substantial incentives are often required to reach break‑even outcomes, particularly for profit‑maximizing ranchers. However, financial incentives alone are insufficient to achieve meaningful conservation outcomes. Briske et al., (2011) reported that structural investments such as fencing or water developments yield limited benefits without effective management, underscoring the importance of managerial capacity.

Greater coordination between Extension and the USDA NRCS offers a promising pathway to enhance adoption. Peterson et al. (2015) highlighted the value of integrating NRCS technical and financial support with Extension’s educational infrastructure through long‑term partnerships. Successful models already exist, such as the Texas Master Irrigator Program, in which over 90% of participants in this educational program report adopting recommended conservation practices (Amosson, 2025). Development of a comparable program for cattle producers could substantially enhance grazing management adoption and outcomes.

### Feedlot systems

Feedlots play an essential role in the U.S. beef industry, with USDA reporting roughly 11.5 million cattle on feed in large commercial operations across the country (USDA, 2026). Beyond beef production, feedlots are an important part of the agricultural economy in states like Kansas, supporting jobs, local businesses, transportation networks, and meat processing while helping sustain rural communities and regional economic growth (Rotz et al., 2019; KDA, 2025).

Challenge: Feedlots pose distinct water quality challenges because runoff and groundwater infiltration can contain high concentrations of nutrients and contaminants, particularly nitrates, phosphorus, and bacteria. Recently, other emerging contaminants have become a concern (Oliver et al., 2020; Mohammadi et al., 2025; Sun et al., 2026). Where sufficient land is available for beneficial application of manure to meet crop nutrient demands, and where adequate separation from freshwater resources is maintained, the risk of water quality impairment is low. In contrast, water quality risks become more pronounced in landscapes with insufficient land for agronomic application (thus resulting in over application of limiting nutrients) and when feedlots and application fields either are close to surface water or overlay shallow groundwater (DeLaune et al., 2012). Because the availability of water for irrigation affects the yield potentials of crops on land-application fields, declining well capacities effectively increase the amount of land required for beneficial use of manure, amplifying the threat posed by manure-borne contaminants if feedlot operators do not observe appropriate agronomic limits on application rates (Marek, 2012; Heflin et al., 2019).

Challenge: Direct water use within U.S. feedlots for drinking, dust control, and facility operations accounts for a small share of total demand compared with the much larger indirect water use from feed production (Rotz et al., 2015, 2019; Asem-Hiablie et al., 2019). Life cycle assessments (LCAs) consistently demonstrate that the apparent water intensity of the feedlot finishing phase is driven primarily by irrigated feed crops rather than on-site feedlot consumption (Castano-Sánchez et al., 2025; Kinghorn et al., 2025). Over the past two decades, direct (blue) water intensity of U.S. beef production has declined due to improvements in animal efficiency and management (Klopatek and Oltjen, 2022). Still, because many feedlots are in water-limited regions, continued efforts to reduce, reuse, and efficiently manage water within feedyards remain critical. Empirical data from commercial operations reinforces this need. For example, at Hy-Plains Feedyard in Montezuma, Kansas, per-head water use peaked during summer months in concert with ambient temperature, underscoring the strong climatic control on drinking water demand and the value of high-resolution monitoring to support feedlot-level water planning (Theurer et al., 2026).

Challenge: Feed‑related management decisions are central in shaping water outcomes across the beef supply chain (Spore et al., 2020). However, regional variations in irrigation availability and cropping systems must be considered. Aquifer depletion poses significant risks for feedlots reliant on irrigated feed and forage. Although grain can be transported economically over long distances, forages’ low bulk density and high-water content reduce their cost-effective hauling distance, increase handling and transportation costs in absolute terms and per unit of feedstuff delivered (Shinners and Friede, 2018). Consequently, dependable local forage production is essential for sustaining cattle feeding operations in the Southern Great Plains.

Concurrently, improvements in irrigation efficiency alone will not solve water‑availability challenges. A growing body of evidence (Pfeiffer and Lin, 2014; Grafton et al., 2018; Sears et al., 2018; Song et al., 2018; Vadjunec et al., 2025; Wang et al., 2020; Xu and Song, 2022) shows that technological gains in water‑use efficiency may accelerate depletion of finite water resources by incentivizing expanded production or increasing consumptive use. Thus, low‑water‑use feed and forages become critical to achieving sustainable water use across livestock production systems.

Finally, agriculture’s economic structure in the Great Plains underscores the importance of water availability for livestock systems. Although irrigated crop production contributes substantially to the regional economy, the livestock sector produces far greater economic returns. In Finney County, Kansas, for example, beef production employs 11.36 times more workers and generates 11.64 times the economic output of irrigated cropping, with profits exceeding crop‑sector returns by a factor of five (Golden, 2023). On a water-use basis, beef production in Kansas generates approximately $165,500 per acre-foot of water used compared to less than $1,000 per acre-foot from irrigated crop production (Guerrero et al., 2013; Golden, 2023). Thus, water is not only an ecological constraint but also a critical economic input underpinning beef production and the broader regional agricultural economy (Hendricks and Sampson, 2022).

Solution: Regulatory frameworks guide nearly every aspect of manure storage, runoff management and containment systems, land application and nutrient management, and other treatment forms (DeLaune et al., 2012). Recent animal waste management innovations for feedlots use advanced systems like anaerobic digestion for renewable natural gas, thermal conversion technologies, and digital monitoring to optimize nutrient applications (Kratzer et al., 2025). These available and economical systems can transform manure from a liability into a resource for energy, nutrient recovery, and carbon sequestration. Wastewater reuse offers another approach to managing feedlot waste. Most feedlots reuse most of their wastewater to irrigate feed and forages (DeLaune et al., 2012); however, advanced wastewater treatment systems are becoming increasingly available and economical, providing new opportunities for beneficial reuse of lagoon wastewater (Kratzer et al., 2025).

Solution: Efficiency improvements in feed and forage systems are essential for reducing the overall water footprint of beef production, particularly in regions where freshwater depletion, such as ongoing declines in the Ogallala Aquifer, threatens the long‑term viability of forage production and cattle feeding operations (Rotz et al., 2026). As shown in Figure 3, multiple opportunities exist to enhance irrigation efficiency and reduce water use.

**Figure 3.**
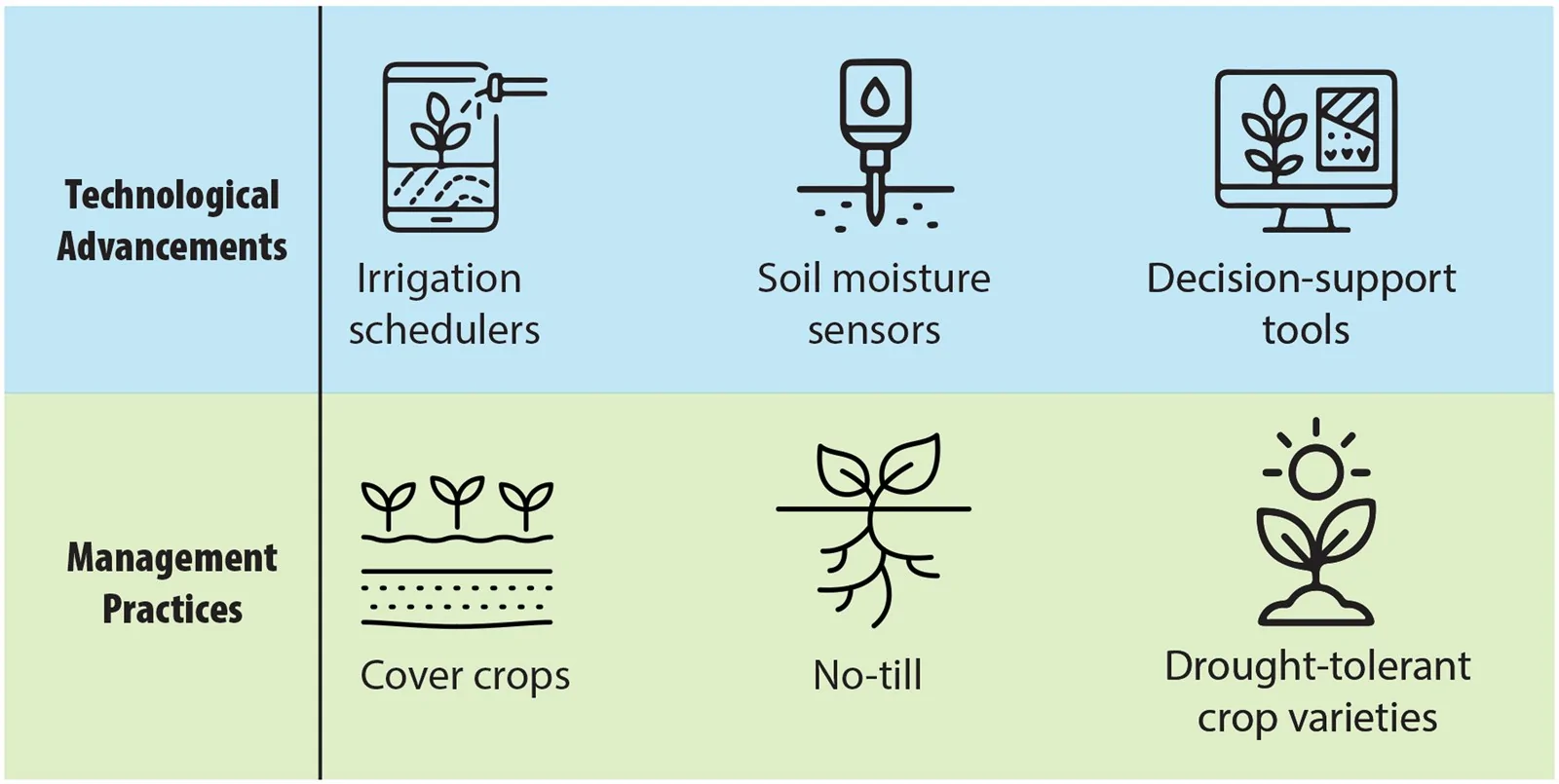
Opportunities to advance irrigation and water use efficiency.

Growing empirical evidence underscores the importance of a systems‑level approach to water management. For example, incorporating byproduct feeds, such as distillers’ grains, reduced water use during the finishing phase by 29% (Spore et al., 2020), demonstrating how feed sourcing choices can reduce water demand substantially without compromising performance. Rotz et al. (2026) highlight that the blue‑water footprint of finishing cattle is highly sensitive to the irrigation requirements of feed crops. Their analyses indicate that substituting irrigated corn and alfalfa with alternative grains and forages can reduce blue‑water use under certain production conditions. However, they emphasize that such dietary changes must be evaluated in the context of broader sustainability metrics, including land use, greenhouse gas emissions, and overall system performance, because tradeoffs among environmental outcomes are common. Consequently, no single diet emerges as the best universal option for finishing cattle across the Ogallala region.

National‑scale assessments also consistently identify feed crop irrigation as the dominant driver of blue‑water use in beef systems. Taken together, these findings suggest that improving irrigation efficiency, optimizing cropping choices, and strengthening regional feed and forage production strategies offer substantial leverage points for reducing beef’s water footprint while sustaining or enhancing productivity (Eshel et al., 2014).

### Overarching challenge and solution

Challenge: Water footprint analyses, showing that beef production requires 80% to 260% more water than other meat sources, have increased both public and scientific scrutiny of beef consumption (Klopatek and Oltjen, 2022). Although national estimates indicate average farm‑gate blue-water use of 2,000 to 2,500 L per kilogram of carcass weight, these aggregated values mask substantial variability across production systems and regions driven by climate, irrigation, and feed sourcing (Rotz et al., 2019). System‑level analyses highlight this heterogeneity, with water footprints differing by 41% to 66% among Texas beef production phases (Menendez and Tedeschi, 2020).

Beef water sustainability is typically evaluated using virtual water footprint or LCA approaches, but differences in system boundaries, functional units, and water accounting methods limit cross‑study comparability (Kinghorn et al., 2025). Cradle‑to‑farm‑gate LCAs, including those developed using the Integrated Farm System Model (IFSM), provide regionally representative estimates across cow–calf, stocker, and feedlot phases. IFSM analyses in Kansas, Oklahoma, and Texas show that blue-water use is concentrated in feed production during the finishing phase, whereas cow–calf systems rely primarily on precipitation‑derived green water (Rotz et al., 2015). However, current assessment methods remain largely static and lack dynamic integration of ruminant nutrition, growth, and temporal system responses, limiting their ability to project future water use under evolving production and climate conditions (Menendez et al., 2023).

Solution: Application of dynamic–mechanistic models captures the complex, time‑dependent interactions that influence water use across cattle production systems, which helps overcome major limitations of current livestock water footprint assessment methods. The Dynamic Beef Water Footprint (DWFB) model uses a system dynamics framework to integrate cattle population dynamics, growth and nutrition processes, and water‑use mechanisms into a series of causal loop diagrams (Menendez et al., 2023). This approach simulates daily water‑footprint behavior and reveals the reinforcing and balancing feedbacks driving water use across cow–calf, stocker, and feedlot phases. By explicitly representing time delays, resource constraints, and management effects, dynamic–mechanistic models generate more realistic and policy‑relevant insights for sustainable water management in beef systems. As livestock data quality and availability continue to improve, adaptable tools like the DWFB model may enhance optimization of management intervention and support efforts to identify animals with superior water‑use ­efficiency (Menendez et al., 2023).

## Knowledge Gaps and Research and Extension Needs

### Watershed and ranch scale data on grazing practice effects

Expanded ranch‑ and watershed‑scale datasets are essential for understanding the benefits of improved grazing systems and for transferring these insights to other regions through enhanced watershed modeling. Long‑term watershed‑scale field studies examining grazing effects on soil water storage, runoff, groundwater recharge, and drought resilience remain limited. Most evidence is derived from plot‑scale experiments, small watersheds (<200 ha), or modeling studies, which often poorly represent large‑scale ranch operations because hydrologic and ecological processes do not scale linearly (Briske et al., 2011, Figure 4). Furthermore, current field studies frequently focus on isolated variables such as infiltration or biomass, limiting inference at the watershed scale.

**Figure 4.**
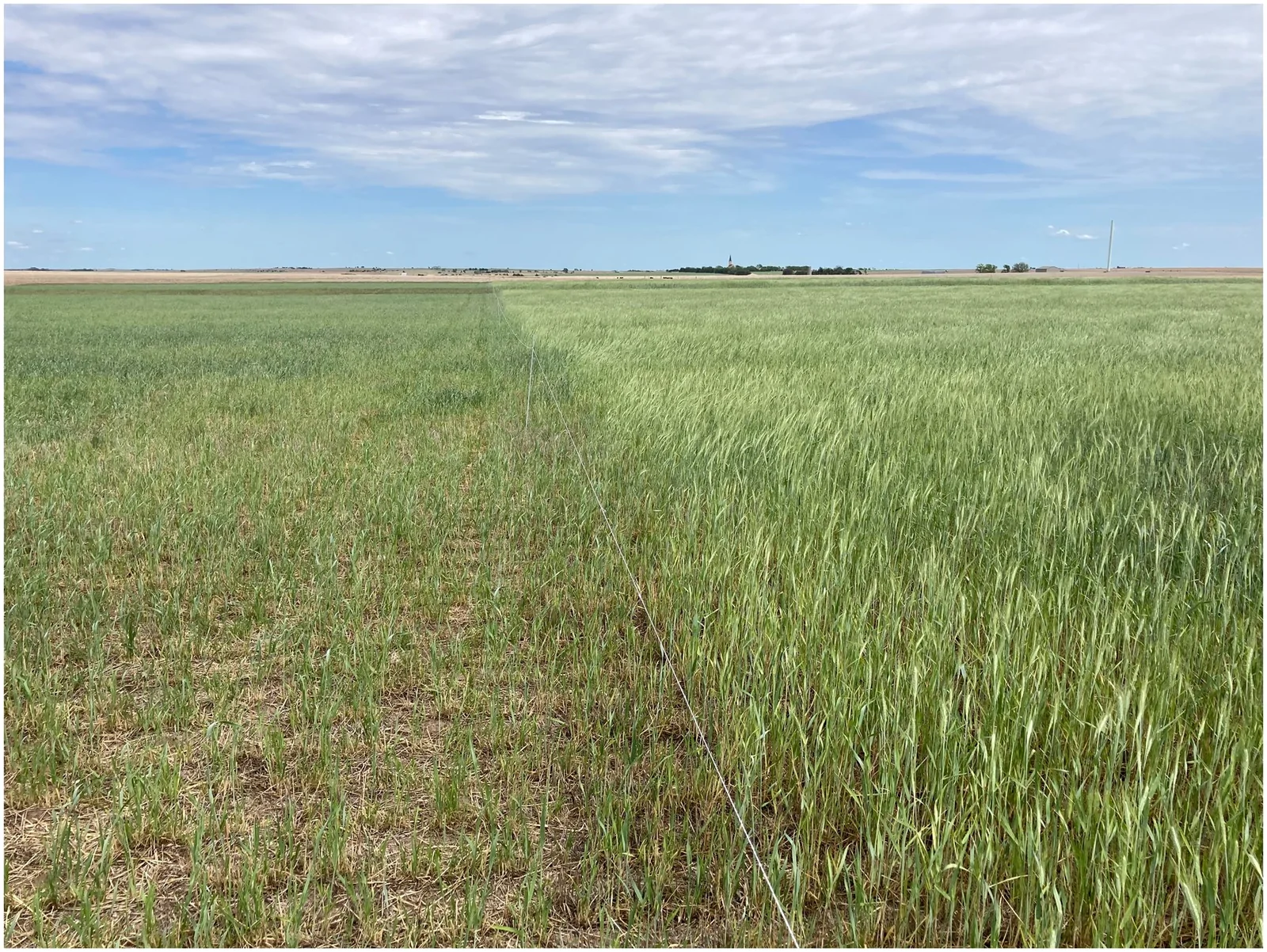
Experimental studies, such as this one in Kansas that examines the effects of grazing cover crops on soil health, should be expanded to include watershed-scale research.

The integration of automated runoff monitoring, continuous soil‑moisture and in‑stream water‑quality sensors, on-animal sensors, and remote sensing platforms and applications offers new opportunities for comprehensive grazing evaluation at ranch and watershed scales. However, these technologies require further exploration and optimization to fully realize their potential (Sudreiani et al. 2023; Chen et al., 2025; Derner et al., 2026). Integration of these high‑resolution and multi‑sensor datasets into artificial intelligence (AI) enhanced modeling frameworks offers additional opportunities to strengthen watershed‑level analyses. Such integration not only improves model performance but supports more robust, regionally transferable assessments of grazing management across a wide range of conditions (Sudreiani et al., 2023; Borankulova et al., 2025).

### Remote sensing, virtual fencing, and other technologies

Recent advances in AI, sensor technologies, remote sensing, and VF offer promising opportunities to enhance grazing management and sustainability (Derner et al., 2026, Figure 5). In particular, AI can support advanced data analytics, decision‑­support systems, and the integration of diverse sensor inputs, enabling more effective livestock monitoring and management (Marchegiani et al., 2025; Derner et al., 2026). However, major knowledge gaps remain.

**Figure 5.**
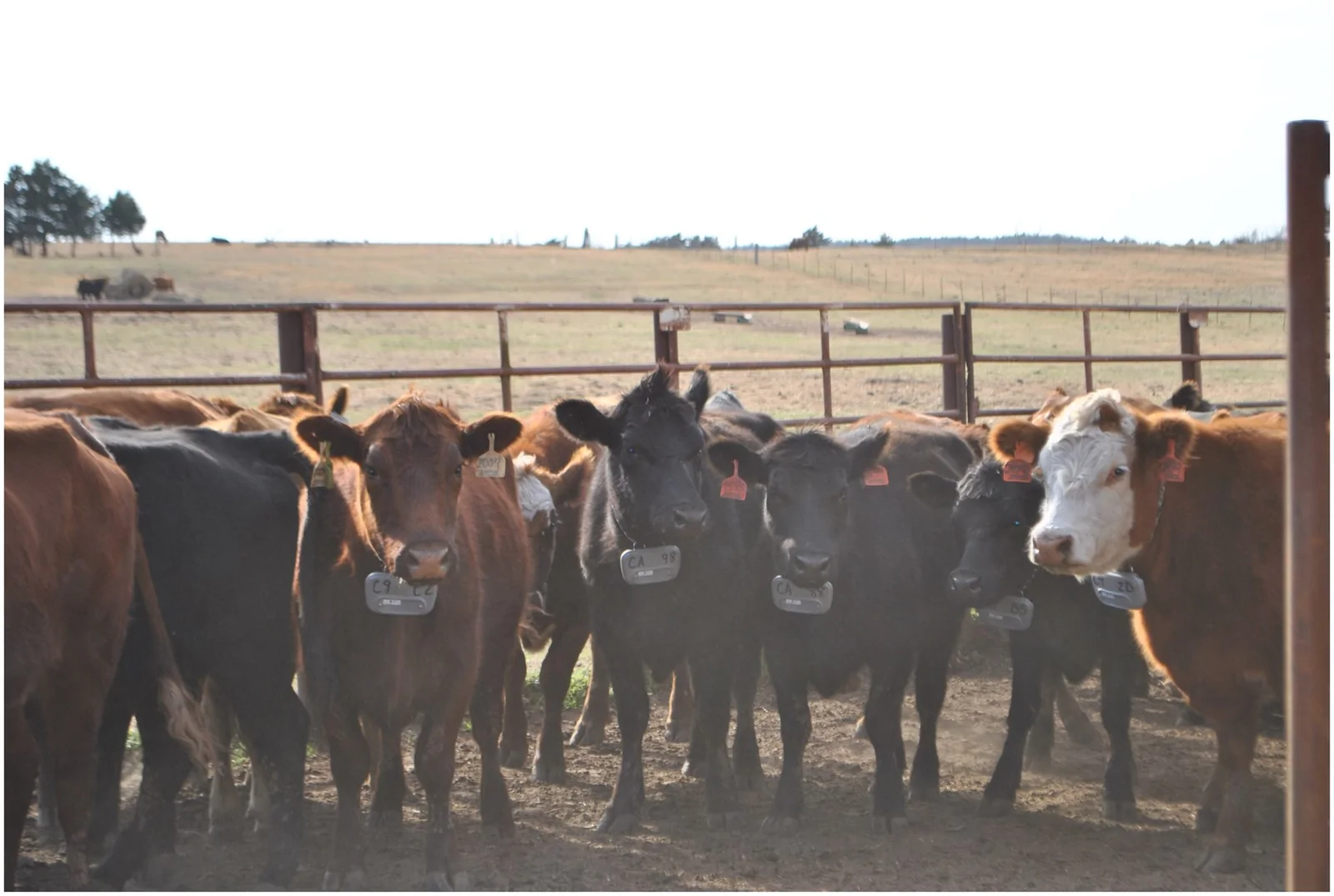
Virtual fencing is emerging as a promising technology for producers in the Great Plains region. However, more research is needed to fully understand and optimize the long-term efficacy of this system.

Improvements in the utility of satellite and other remotely sensed imagery for estimating total standing dry matter is needed to guide grazing at the pasture and farm level (Ogungbuyi et al., 2023). Concurrently, advancements of VF and integration with fine-scale remote sensing of forage could advance adaptive management of grazing land social-ecological systems (Derner et al., 2026). Long‑term effects of VF on ecosystem services (Murray et al., 2025), animal behavior and health, and rancher economic viability, particularly for small‑scale producers, are poorly understood. Research is needed to improve VF reliability, identify sources of performance variability (Murray et al., 2025), mitigate failure modes, and facilitate broader adoption. High costs and limited technical expertise continue to hinder technology uptake (Marchegiani et al., 2025), and the development of scalable, secure data systems is essential to support these emerging technologies.

### Feedyard water quality, use, and reuse

Additional research is needed to develop practical, realistic, and cost‑effective livestock‑water reuse strategies (Parker et al., 2000, Figure 6). Priorities include defining appropriate drinking water and operational reuse standards and evaluating effective feedyard-scale treatment technologies. Understanding pharmaceuticals’ fate and treatment and other contaminants of emerging concern in livestock waste systems is also essential for protecting animal and environmental health (Theurer et al., 2026). Direct evidence linking water quality, particularly microbiological and organic chemical contaminants, to carcass traits and meat quality is largely missing. Understanding this is key to developing baseline drinking water quality guidelines for water reuse technologies (Theurer et al., 2026).

**Figure 6.**
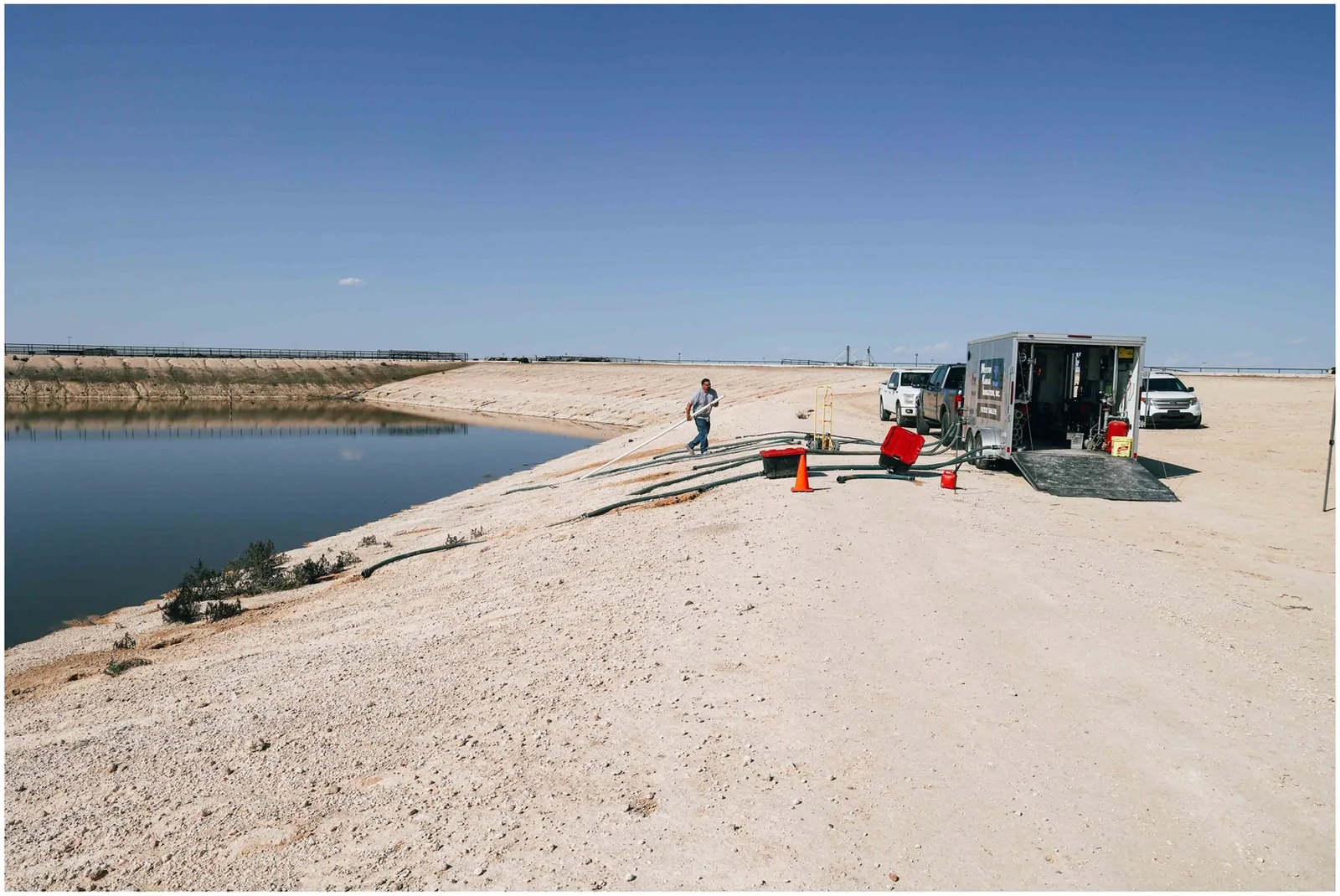
Experimental systems like this one in western Kansas explore the option of recycling lagoon water into drinking water for cattle.

#### Advancing technology for livestock wastewater reuse

A promising option for lagoon water reuse is anaerobic membrane bioreactors (AnMBRs) which couple waste treatment with energy recovery while producing high‑quality effluent suitable for reuse. Pilot‑scale AnMBRs operated under ambient conditions have shown effective removal of organic matter and facilitation of nutrient recovery, indicating strong potential to reduce freshwater demand and improve lagoon management (Kratzer et al., 2025).

### Drought-tolerant forages, feedstuffs, and cropping systems

Strengthening feed system resilience will be central to sustaining beef production in water-limited regions. Continued development and adoption of drought-tolerant crops, combined with better forecasting, improved crop management, and more efficient irrigation, offer practical pathways to reduce water demand while maintaining feed availability. Feed production remains the largest opportunity to influence the overall water footprint of beef, and adjustments at this stage can yield meaningful gains (Rotz et al., 2026). Shifting acreage from highly irrigated corn and soybean systems to less water-intensive feed crops like sorghum and wheat has been shown to reduce blue-water use without proportionally reducing output, particularly when combined with improvements in irrigation efficiency and management (Castano-Sánchez et al., 2025). National LCAs reinforce this systems perspective, showing that water use embedded in feed production far exceeds direct cattle water use and highlighting the importance of integrated crop–livestock strategies that align feed sourcing with regional water availability (Asem-Hiablie et al., 2019).

### Socioeconomic and policy dimensions

Adoption barriers, including insurance and financing limitations, regulatory uncertainty, and misaligned policy incentives continue to constrain on-the-ground change, despite an increasing recognition of the need for water conservation. Prior research on producer-perspectives has largely focused on irrigated crop producers, with less attention given to motivations, constraints, and decision frameworks of beef producers, even in regions where cattle production relies on groundwater-dependent feed systems (Aimpoint Research and Kansas Water Institute, 2024). A more comprehensive understanding of producer incentives, risk tolerance, and operational priorities is needed to design effective and durable conservation strategies.

Modeling of the longest-running Local Enhanced Management Area in Kansas demonstrates that producer-led irrigation reductions, combined with shifts to less water-intensive cropping systems, reduced blue-water use by 28%, maintained economic viability, and incurred only modest yield penalties (Castano-Sánchez et al., 2025). However, evidence from the Ogallala region highlights persistent adoption challenges. A 2018 irrigator survey found that while producers strongly value water conservation for drought risk management and community sustainability, they often believe they have maximized their efficiency; therefore, adoption of advanced irrigation management tools remains limited (Lauer and Sanderson, 2020). Time constraints, limited technical support, benefit uncertainty, and limited trust in new technologies continue to hinder broader uptake, underscoring the importance of policies and programs that align conservation goals with producer capacity and operational realities.

## Conclusions and Recommendations

Landscape-level management decisions shape water sustainability in U.S. beef cattle production. Across grazing lands and feedlot systems, the dominant water-related challenges include increasing drought frequency and intensity, water quality impairment linked to runoff and nutrient and bacterial loading, declining water availability for irrigated feed and forage production, and growing public scrutiny driven by simplified water footprint metrics. The literature demonstrates that adaptive grazing management, riparian protection, strategic feed and forage sourcing, and integrated crop–livestock systems can substantially improve water outcomes while maintaining or enhancing production resilience. However, regional outcomes vary, underscoring the limitations of single, aggregated water footprint values and the need for context-specific, systems-based assessments that reflect local climate, hydrology, and management realities.

Technological efficiency gains alone cannot achieve long-term water sustainability. While advances in irrigation efficiency, animal performance, and waste management are necessary, adoption of proven practices is strongly constrained by economic risk, delayed returns, labor requirements, land tenure, and policy misalignment. Coordinated Extension programming, incentive-based conservation frameworks, and producer-led governance approaches are therefore essential for scaling water stewardship. Emerging tools like remote sensing, high resolution monitoring, and VF show promise for reducing adoption barriers, but their effectiveness depends on improved data integration, model representation, and long-term field validation at ranch and watershed scales.

Based on these findings, priority recommendations include:

Expand long-term ranch and watershed-scale field studies to quantify the hydrologic, water quality, and drought resilience impacts of grazing and riparian management across diverse regions.

Enhance water footprint and watershed models to better represent management intensity, temporal dynamics, and tradeoffs among water, economic, and environmental outcomes.

Advance research and deployment of cost-effective feedyard wastewater reuse and treatment technologies, including evaluation of contaminants of emerging concern.

Strengthen drought-resilient feed and forage systems development through breeding and genetics, crop selection, irrigation management, and integrated crop–livestock strategies.

Align Extension, policy, and incentive programs to reduce adoption barriers and support durable, scalable improvements in water stewardship across the beef supply chain.

## Author Biographies

**Kevin L. Wagner** serves as the Director of the Oklahoma NSF EPSCoR Program and Director of the Oklahoma Water Resources Center at Oklahoma State University. As Director of the Water Center, he provides leadership and administration of the Center’s water research, outreach, education, and grant programs; leads Center efforts to increase engagement with the water resources community across Oklahoma and the United States; and facilitates development of interdisciplinary teams to address high priority water resources issues. As Director of the EPSCoR Program, he provides leadership and administration of a large multi-institutional and multidisciplinary research team aimed at finding Socially Sustainable Solutions for Water, Carbon, and Infrastructure Resilience in Oklahoma.

**Susan T. Metzger** serves as director of the Kansas Water Institute (KWI) and KCARE. Susan is the Director of Strategic Interdisciplinary Program Development for Kansas State University. Susan has broad experience representing Kansas State University and Kansas agriculture. Before her work at K-State, she was the Deputy Secretary of the Kansas Department of Agriculture, where she served the farmers, ranchers and agribusinesses of Kansas and assisted in the development and delivery of tools to grow the state’s agricultural economy. Prior to that, she was the Chief of Planning and Policy for the Kansas Water Office, where she was the team leader in the development and implementation of the Long-Term Vision for the Future of Water Supply in Kansas.

**Brent Auvermann** is based in Amarillo, Texas, where his extension and research programs focus on environmental quality and manure management associated with confined livestock production systems. He specializes in water quality, air quality, manure and mortality management, and biomass energy as they relate to beef and dairy production in confinement. His work emphasizes feedyard and dairy manure management, compost production and land application, dust and odor abatement, feedyard dust characterization, and air pollution policy analysis. Dr. Auvermann serves as an adjunct faculty member in the College of Agriculture and Natural Sciences at West Texas A&M University.
